# Feedback in Music Performance Teaching

**DOI:** 10.3389/fpsyg.2022.891025

**Published:** 2022-06-20

**Authors:** Gary E. McPherson, Jennifer Blackwell, John Hattie

**Affiliations:** ^1^The University of Melbourne, Parkville, VIC, Australia; ^2^University of Hawai‘i at Mānoa, Honolulu, HI, United States

**Keywords:** feedback, music performance, performance teaching, visible learning, autonomy, self-regulated learning

## Abstract

The purpose of this article is to provide one prominent perspective from the research literature on a conception of feedback in educational psychology as proposed by John Hattie and colleagues, and to then adapt these concepts to develop a framework that can be applied in music performance teaching at a variety of levels. The article confronts what we see as a lack of understanding about the importance of this topic in music education and provides suggestions that will help music teachers refocus how they use feedback within their teaching. Throughout the article, we draw heavily on the work of John Hattie and his colleagues whose explanations on all facets of feedback, but especially those forms of feedback that are focused on ensuring students understand “where to next”—have had a huge impact on school education through various publications.

## Introduction

As we began writing this article, we cannot think of any area of human pursuit where feedback isn't an integral feature of learning and further development. We rely on feedback: from self-observation of our own efforts at mastering a new concept or skill, through to the comments and suggestions from our teacher, coaches, peers, and supervisors that help us monitor and improve our performance, build our confidence and cope with even the most mundane learning situation. It is therefore surprising that there is so little understanding of the concept in music beyond the basics of describing the process of providing information back to a learner that can help that person master some sort of musical skill. The emphasis is often on teacher to student interactions, which negate other important forms of feedback. Observing or analyzing our own performance, moving from student to teacher or peer to peer, and understanding the various forms of feedback that can work to enhance learning, are essential if teachers are to appreciate more fully the power of feedback in music learning.

The purpose of this article is to provide one prominent perspective from the research literature on a conception of feedback in educational psychology as proposed by John Hattie and colleagues, and to then adapt these concepts to develop a framework that can be applied in instrumental/vocal music teaching for a variety of levels of student ability. First, we define feedback according to the work of Hattie and colleagues. We then describe the types and levels of Hattie's Visible Learning framework in detail. We provide some guidance on how to give feedback at an appropriate level and considering other actors in the feedback process beyond the teacher. We then turn to other important considerations for feedback, including questioning techniques, issues surrounding praise, external rewards and performances-based feedback, and the importance of student reception of feedback. Finally, we discuss how these feedback processes might be adapted in music performance teaching contexts.

One of the remarkable features of feedback is that it is not only among the more significant influences to improve performance but is also one of the most variable. For example, a major review by Kluger and DeNisi (1996) showed that 1/3rd of feedback is negative. It is therefore not a simple matter of providing feedback, or providing lots of feedback, but having a deeper understanding of how effective feedback works, when, and for whom. Our emphasis will be to confront what we see as a lack of understanding about the importance of this topic in music education and provide suggestions that will help music teachers optimize their performance teaching. We draw heavily on the work of Hattie and his colleagues (especially, Hattie and Timperley, 2007; Hattie and Clarke, 2018; Brooks et al., 2019a; Wisniewski et al., 2020) whose framework for explaining all facets of feedback—but especially those forms of feedback that are focused on ensuring students understand “where to next”—has had a huge impact on school education through various publications (e.g., Hattie, 2012; Hattie and Clarke, 2018; Hattie and Zierer, 2018; Hattie et al., 2021).

## What is Feedback?

When asked to describe what is meant by feedback, teachers will often explain that it is a way of giving comments on what is being learned, answering student questions, giving an instruction or criticism, confirming that the learner is taking the right approach (or alternatively not taking the right approach), explaining the pros and cons of working in a certain way, or providing an assessment of work relative to some sort of standard or benchmark. But when the same question is asked of students, they will often explain how feedback helps them know where to go next, and if they do not see any “where to next” feedback they will even go so far as to suggest that they did not receive any feedback (Hattie and Clarke, 2018). This contradiction between how teachers view feedback and what students feel is the most valuable form of feedback for them is why this topic is so important in all forms of teaching, and especially music, where lessons often include students who have chosen to participate in elective music classes, choose to participate in large ensembles, or are engaged in a master-apprentice learning context that is typical within one-to-one studio performance teaching settings. Hattie and Timperley (2007) define feedback as “information provided by an agent (e.g., teacher, peer, book, parent, self, experience) regarding aspects of one's performance or understanding” (p. 81). This conception is broad enough to include a variety of types of feedback as well as a variety of actors in the feedback process and is the basis for how we conceptualize feedback for music contexts here.

### A Conception of Feedback

In the research literature, feedback was seen for many years through the lens of how to reduce discrepancies between what a learner can do now and what they strive to achieve or master. Essentially, views centered around feedback as a “consequence of learning” and as a means of “closing the gap” by providing corrective information to clarify an idea, receive encouragement, or evaluate the correctness of a response (Sadler, 1989). More recently, feedback has been conceptualized more broadly (Hattie and Timperley, 2007; Wisniewski et al., 2020). One of the most prominent educational scholars on feedback is John Hattie, who has proposed a conception of feedback that is multifaceted, including a variety of potential sources, types, and levels of feedback.

Instead of information that is provided by a teacher to a student, the emphasis has shifted to how different agents who provide feedback (e.g., teachers, parents, peers, online and printed resources, or even the person who is receiving the information) provide information from different perspectives and levels of cognitive complexity, and the degree to which the recipient hears, understands, and actions the feedback. Learning is also inherently emotional, as learners must work through their shortcomings to improve performance. This means that feedback must be delivered in environments that consider student affect. Hattie et al. (2021) emphasized the importance of strong student-teacher relationships, including warmth, trust, and empathy. With positive relationships, students can see the learning environment as a safe space to grow, where mistakes are viewed as an opportunity to learn, and thus feedback is a welcome component of growth and understanding. In the following sections we describe the main facets of this approach and suggest music related examples to explain how they might be applied in music teaching and learning settings.

### Visible Learning

Hattie's *visible learning* (Hattie, 2011; Hattie and Clarke, 2018) is a program of professional development focused on techniques that empower teachers to become evaluators of their own teaching who see learning through the eyes of their students. This approach advocates three types of feedback for enhancing learning and achievement:

- “*Feed back”* compares a learner's current state with previous performance, such as pointing out improvements in the playing of repertoire since the previous lesson or attempt at the passage.
- “*Feed up”* is focused on the present and compares what a learner can do with a desired target state. Examples include critiquing a student's current level of playing and comparing this with an ideal performance once the work has been mastered.
- “*Feed forward”* focuses on illustrating the desired target state. Because it is aimed at what a student will be able to do in the future, it is the most desired form of feedback for students.

**Figure 1:**
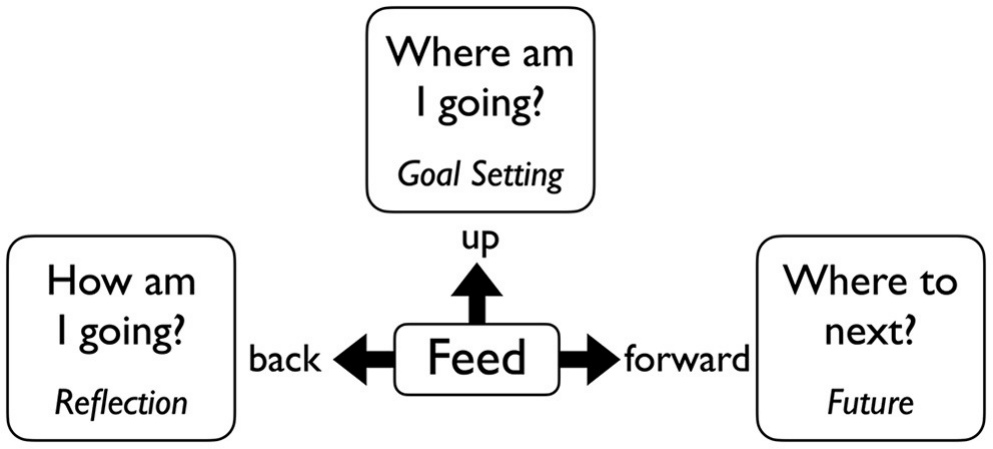
Visual depiction of feed back, feed up, and feed forward.

The three forms of feedback outlined above emphasize past, present, and future perspectives and are most successful when they enable students to understand how they are going now, where they are going next, and how they might get to the next level (see Figure 1).

### Cognitive Complexity

According to Hattie and colleagues, these three questions can be addressed with feedback at multiple levels of cognitive complexity (Wisniewski et al., 2020). While, not currently reflected in the music literature, these processes map well onto music learning contexts and can provide important guidance for how we might conceptualize the feedback process.

*Task level feedback* refers to how a teacher might give feedback about the content of what is being learned, facts associated with learning this task, or even how well the task has been completed and understood. Providing correct or incorrect feedback can be powerful at this stage, especially when it is immediate. In music, this might entail telling a student they are playing a passage out of time, that they've improperly placed the sharps in a written key signature, or that they've appropriately played an exercise in tune.

*Process level feedback* is information more about the strategies or processing by the student. The teacher listens to the strategies that the student claims they are using, provides information about checking, correcting, or alternative strategies, helps the student in error detection, and showing them where they could have sought help and further feedback. The key component of this level is that the teacher is working with the student in providing feedback.

*Self-regulation feedback* occurs when the emphasis is on how a learner receives information about how to monitor and control their own use of strategies for completing a task and develops their skills to self-evaluate their own progress and the confidence needed to continue their own development. This level of feedback is based in the broader construct of self-regulated learning, which Zimmerman and Schunk (2011) define as “the processes whereby learners personally activate and sustain cognitions, affects, and behaviors that are systematically oriented toward the attainment of personal goals.” (p. 1). By setting personal performance goals, learners create feedback loops through which they can monitor their effectiveness and adapt their strategies for future learning. A good example is when a student engages in self-evaluation, critique, seeking help, trying new strategies, and transferring their skills and knowledge to new or transfer tasks. The key component of this level is that the student is taking the initiative, but it does not preclude teachers supporting, prompting, and evaluating students' self-initiated regulation of their learning.

There is another level in Hattie's framework regarding *self-level feedback*, but it is of such low value to the learner's improvement that it is best not included as a worthwhile level of feedback for enhancing learning. Comments aimed at the learner personally rather than their accomplishment of a task are far less effective than corrective feedback for effecting change in the learner (Wisniewski et al., 2020). In self-level feedback the focus is on personal characteristics, such as when a teacher praises a learner, rather than the content of what they are doing or the strategies they have employed. This might occur when a teacher praises (or criticizes) a student personally rather than praises or criticizes an aspect of their performance. We return to praise below.

The research literature shows that task level feedback is the most common in learning situations, but the power of feedback can be high at the task, process, and self-regulation levels, and as the student becomes more proficient it is valuable for teachers to provide more process and self-regulation feedback. At all levels, the most effective feedback is focused on “where to next” (Hattie and Timperley, 2007; Wisniewski et al., 2020). As Wisniewski et al. (2020) show, “feedback needs to focus on the appropriate question and level of cognitive complexity; if not the message can easily be ignored, misunderstood and of low value to the recipient” (p. 2). As explained below, this means that praise, coercion, and rewards are far less valuable and effective for a music learner than corrective feedback that focuses them on mastering new skills and techniques (Wisniewski et al., 2020).

## Effective Feedback

For feedback to be effective, it needs to be pitched at an appropriate level for the learner. It should also aim to move them upward through the various levels required to achieve mastery of the task or skill being learned. In general, feedback that is given at or just above the level the student is working at and focused on the task, process, and self-regulatory mechanisms required to develop mastery should be the aim. To do this, teachers should avoid repetitive comments that merely identify errors in performance, and instead provide a clear indication to the student about “where to next”. Obviously, students within the same class can be processing and learning at different levels; thus, feedback needs to be tailored to where each student is in the learning levels, as what might work for one student may not work for another. Some students can be reluctant to move away from receiving and acting on feedback at the task level, as this is more easily processed, but can led to more mechanical playing, less depth of understanding, and lower levels of musicality.

Earlier in this article we discussed three forms of feedback: “feed up”, “feed back”, and “feed forward”. When implemented appropriately in teaching, these three forms of feedback emphasize *past, present*, and *future* perspectives. They are most successful when they lead to students understanding how they are going now, where they are going next, and how they might get to the next level or reach defined personal goals (McPherson and Hattie, 2022; see Table 1).

**Table 1:** Levels of feedback.

**Levels of feedback**				
		**Task**	**Process**	**Self-regulation**
Perspectives on feedback	Past (“feed back”)	What progress has the learner made in learning their repertoire and technical skills?	What progress has the learner made on learning new repertoire? Is there evidence of improvement?	What progress has the learner made in developing self-regulated practice strategies?
	Present (“feed up”)	What goals did the learner reach? What musical content did the learner understand?	How did the learner learn the passage? Is there evidence of effective practice strategies?	What self-regulated practice strategies did the learner apply?
	Future (“feed forward”)	What goals should be set next? What repertoire or skills should be learned next?	What practice strategies should the learner apply next?	What self-regulated practice strategies should the learner apply next?

*Adapted for instrumental/vocal music practice from Hattie and Zierer (2018, p. 89)*.

## Other Actors in the Feedback Process

Up until this point, we have considered feedback according to the actions or comments provided from a teacher to the student. However, a singular focus on this perspective can be flawed, especially when it reinforces notions that the teacher is the person most responsible for providing a comprehensive analysis of where the student is up to and where to next. It should be clear from the three levels that the more the teacher is in “control” of the teaching then the more the feedback must stay at the task-level and thus hamper the growth of the student. It is the learner who is in the best position to answer questions about whether they feel they have achieved their goals, the extent to which they have understood what is being taught, and how helpful they regard the suggestions or method for improving their performance that have been provided by the teacher. Learner to teacher interactions are therefore of fundamental importance given that very little of what happens in a lesson is observable. Most of what we do is not immediately apparent and therefore needs to be made visible through student action (Hattie, 2011; McPherson and Hattie, 2022).

Every time teachers make themselves aware of what a student is thinking, how the student would evaluate the effectiveness of a method they aiming to master, or how the student feels about themselves, they make learning visible to both themselves and the student. And together, the teacher and student are also far better positioned to jointly negotiate a pathway forward (McPherson and Hattie, 2022). An ideal learning environment is one where mistakes are seen in a positive light, with quality feedback being the mechanism to help the learner grow from errors and misconceptions. Feedback feeds on errors and not knowing, and no student comes to a learning situation already knowing everything. While, it is also important to highlight what the student is doing correctly in order to keep them going on the right direction, thinking of feedback as a more neutral process where students receive information aimed at helping them improve is perhaps healthier than conceiving of feedback as “positive” or “negative.” This reinforces the importance of developing teacher-student relationships that lead to trust in students acknowledging errors and misunderstandings, but also strengths, which makes the feedback more effective (Hattie et al., 2017). We see this play out in the attitudes and the comments of performance teachers, such as those of renowned violin teacher Brenda Brenner, who said the following about student errors:

I want them to make mistakes, and I try to use them as a launching pad for our conversations about what they should be thinking about when they are making these decisions musically. Mistakes are just information that will help them get better (Blackwell, 2022, p. 6).

Thus, when students and teachers see musical errors as a launch pad to improve their musical skills, the “where to next” of musical learning becomes visible.

## Other Considerations for Feedback

### Questioning

Teachers ask questions as a way of determining their student's current level of knowledge, but also to gain an understanding of how well they are mastering new information. Teachers can probe a student with questions such as “Tell me what you're going to do first to master this new passage”, “Why was that performance better than your previous performance?”, or “How can you change what you've written in your composition to convey the mood of this section more clearly?” Hattie and Clarke (2018) show that probing questions like these allow more to be revealed, so making statements such as “What do you mean by…?” helps get to the heart of a student's current understanding.

From another perspective, Nystrand (2006) has shown that the more powerful questions students can ask themselves, their teacher, or their peers, are “impact questions”; that is, questions that have no pre-specified answer (e.g., “What would happen if…?”), where student responses are used in follow up questions by the teacher (“Has hearing David perform that section changed how you would approach your own performance?”), or where a student response is used to modify further discussion (e.g., “I agree with Mary that this section works better if phrased by…”).

### Feedback vs. Praise

As humans, we all enjoy receiving praise. Our tendency is to interpret the praise we receive as an indication that the person likes something about us or what we are doing. But how effective is praise for motivating learning and helping a learner move to the next level? Within learning contexts, praise is rarely an effective means for improving performance (Lipnevich and Smith, 2008) and can actually interfere with learning because it is something that is directed at a person or their accomplishments and so will often be remembered more than any substantive feedback (Hattie and Clarke, 2018). Imagine you receive feedback relating to the musical task you are doing and at the same time receive praise about you or your effort on the task. When students are later asked to recall the feedback, they tend to only recall the praise—hence, praise can dilute worthwhile feedback. Even more disconcertingly, studies comparing students who are and are not given praise by their teacher provide strong evidence that no form of praise has a positive impact on learning if it is provided to encourage a desired behavior (Skipper and Douglas, 2012). Person praise has also been found to be outright detrimental (especially with regard to persistence after failure), whereas process praise is no more effective than objective feedback. So, giving praise for being helpful, completing an activity, performing well against certain criteria or for engaging with, but not necessarily completing an activity, can all have a negative impact on learning outcomes. Moreover, the authors noted successive failures were not helped by any type of feedback, which is important for understanding the necessity of careful scaffolding so that students will persist with their efforts and not experience negative affect. This is not suggesting that we need to be negative- to the contrary- person praise can be the essence of building relationships, but praise should not be confused for or mixed with feedback. It is therefore important not to give praise in moments when students should expect feedback, such as directly after a performance trial. As music teachers, we need to use praise in ways that do not mix feedback about what is being learned with any personal qualities of the student.

### External Rewards

External rewards are sometimes used in music teaching settings as a form of feedback for what is seen as positive learning behavior. However, it is now well-established in virtually every learning and work environment that external rewards erode achievement and motivation (Deci et al., 2001; Hattie and Clarke, 2018; Evans and Ryan, 2022). External rewards such as giving stickers, awards and even pocket money to complete practice can seem innocent enough, but throughout academic learning and in music these types of external reinforcements have consistently been found to reduce motivation over time. An example is the study by McPherson and colleagues (McPherson et al., 2012) of young beginning instrumentalists: not one of the children who were given rewards or incentives to learn (e.g., pocket money) continued learning beyond the first year of study. This is because rewards and punishment are usually ineffective at changing a learner's behavior (Deci and Ryan, 2004). Moreover, extrinsic rewards can actually reduce a students' preexisting intrinsic motivation to engage in music, replacing their desire to learn music for its own sake.

As we have seen in the previous sections, praise and extrinsic rewards can have a detrimental impact on both learning and motivation. What types of feedback, then, will impact positively on learning and motivation? To answer this question, it is important to realize that from the meta-synthesis studies conducted by Hattie (Hattie and Timperley, 2007; Hattie, 2009; Hattie and Zierer, 2018) high effect sizes have been found for feedback on student achievement in academic learning. These effects are much higher than other factors, such as the time devoted to a task, practice and homework, teacher questioning, and peer influences. However, they can also be considerably lower, depending on the type of feedback. By far the most powerful effect sizes are attributed to feedback that helps students develop more efficient and effective processing strategies and understandings (Hattie and Timperley, 2007).

### Student Reception of Feedback

It is self-evident that no feedback will be effective unless it is heard, understood, and actioned by the student. While, there is much research on the provision of feedback, there is less about how students receive the feedback. What we do know is that when students are asked about what they mean by feedback, it is most often expressed in terms of “where to next” and “how to get to the next level of performance.” In this way, and as stressed earlier, students are more forward focused whereas teachers tend to be more backward in their comments.

Skilled teachers understand the importance of inviting students to comment on the feedback they provide for two major reasons: (i) to ask if they heard and understood the feedback, and to hear whether they actioned or can action the feedback, and (ii) to assess for themselves whether their feedback was effective and thus readjust how and when to give feedback to ensure it is heard, understood, and actionable.

Contemporary conceptualizations of feedback explore how students can be encouraged to actively seek out, create, provide, discuss, and apply feedback to progress their own learning (Brooks et al., 2021a,b). This newly emerging focus challenges notions of a teacher setting work and providing highly directive feedback, and instead stresses ways to improve the learner by developing their capacity to self-regulate (Brooks et al., 2019b, 2021a,b; Casas-Mas et al., 2019; López-Íñiguez and McPherson, 2020, 2021; Mandouit, 2020).

### Issues With Person-Based Feedback

If you watch music performance teachers, you will hear different types of praise and criticism with comments by the teacher such as “You're very talented” or “You're really good at this”. However, this type of feedback has virtually no impact on learning because the focus is on the individual and not about the learning process. Too much ongoing praise can lead to a reduction in some learner's willingness to try harder. In addition, too much criticism can lead students to develop a negative self-concept for performing, especially when they feel their abilities are not being fully recognized. Indeed, intrinsic motivation can be undermined in some highly motivated students when teachers use praise as a form of extrinsic motivation. Importantly, praise and criticism can be problematic if they reduce a student's focus on what they are trying to master.

Research evidence in education and music does not suggest that teachers should never give any praise or criticism, however. As an example, Duke and Simmons (2006) studied the studio lessons of renowned artist-teachers and found there were infrequent, intermittent, and often unexpected instances within lessons when these teachers provided negative—perhaps better termed corrective—feedback that was clearly directed to improving the quality of the performance. Importantly though, there were also other moments in the lesson where “intense praise” was used to convey how excited they were that the student had been able to master a new learning challenge. Blackwell (2020, 2022) found that studio teachers use a form of “neutral” feedback (where negative and positive aspects of the student performance are presented unemotionally side-by-side), and that this can be an effective way of getting the students to trust their teacher's feedback as an honest or fair evaluation of their playing. This does sometimes manifest as a form of praise, but not an effusive one. These music studies are consistent with the educational research showing that “less is more” regarding when and how often a teacher should provide self-feedback and studies showing that constant praise can act as a diluter when it is mixed with feedback about the work (Hattie and Zierer, 2018).

## Giving and Receiving Feedback

Many music performance teaching contexts have been criticized for being overly teacher-centric and promoting passivity in students. For example, the master-apprentice studio teaching model has been criticized as overly focused on teacher directed and authoritarian styles of teaching (McPherson and Hattie, 2022); something that Hyry-Beihammner (2010) suggests has been romanticized because of its perceived emphasis on the intimate personal relationship between teacher and student but is too often focused on the authority of the teacher with an undue emphasis on imitation learning (Nielsen and Kvale, 1997). In the best examples of studio teaching, students can be inspired to attain levels of performance excellence that are well beyond what they might have expected for themselves, gain dispositional qualities, techniques, and understandings that are fundamental to their future success as performing musicians. They also become personally connected with a mentor whom they admire and who can help them develop a sense of their musical future selves.

Less effective studio teaching is characterized by teachers who talk to their students in short utterances about the previous or upcoming actions concerning how the music should be performed and provide few opportunities for their students to reflect on what they have done well or where they have gone wrong (Rostvall and West, 2003). The problem with this later type of teaching dynamic is the undue emphasis on a highly experienced music performer imparting their knowledge to an often passively receptive learner (Blackwell et al., 2020). This hierarchical and asymmetric pattern of interaction leaves little room for students and teachers to discuss and reflect on the process, provides few opportunities for the student to develop musical independence, and results in few opportunities for input from the students.

As suggested above, effective teaching involves more than a flow of information from teacher to student. Educational research has asserted that feedback should involve a two-way process and that some of the most powerful exchanges occurs when students give their teacher feedback about the impact of their teaching (McPherson and Hattie, 2022). Effective teachers listen to student comments, analyze whether they have been able to connect what they have been learning with new material, and assess whether they have gained in confidence and enjoyment of the learning process.

## Adapting Education Research on Feedback to Musical Learning

With the above framework in mind, we now move to providing comments on how teachers of music can maximize the effectiveness of feedback in their own music teaching. To begin, we are reminded of Sinek's (2009) powerful comment that there are only two ways of changing behavior: we can either manipulate a learner's behavior, or inspire the learner to strive even harder (see McPherson and Hattie, 2022). As teachers, when we focus on telling students what we want them to learn and what to do to achieve our goals for them, we are manipulating their behavior. Succeeding in a future performance and mastering a new technique are goals we set that are external to the learner. In contrast, when we take the time in our feedback to explain why we are introducing a new idea, strategy, or technique, we are contextualizing learning and placing the emphasis on the purpose, cause or beliefs associated with this learning task. We know that learners are better positioned to thrive when they understand why they are being asked to take on a new challenge, especially when this information is presented in parallel to information that indicates how mastering a task can allow them to tackle other technical and musical challenges. Most importantly though, true understanding of the purposes of learning helps bolster the learner's personal beliefs in themselves and what they can accomplish into the future, which makes feedback more meaningful and actionable (McPherson and Hattie, 2022).

### A Matrix of Feedback for Learning

**Figure 2:**
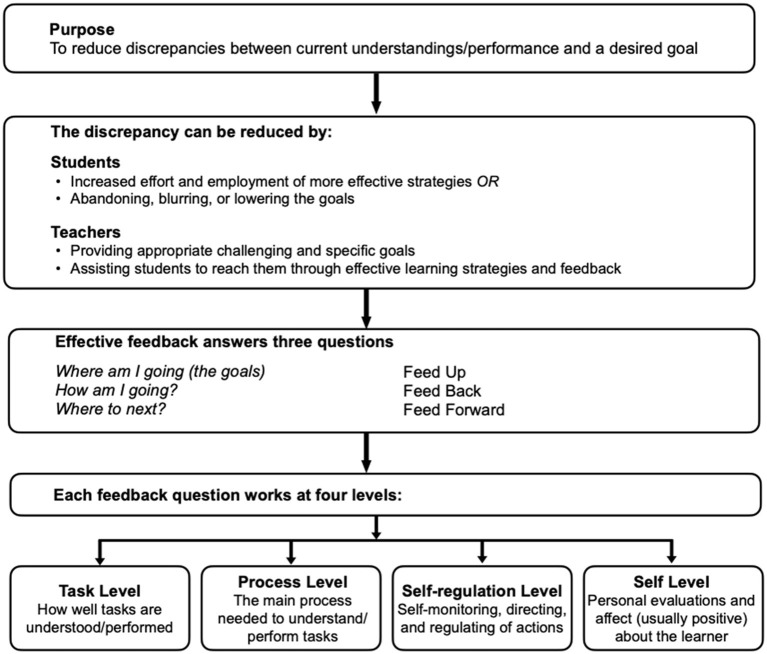
A model of feedback to enhance learning. Reprinted with permission from Hattie and Timperley (2007, p. 87).

Figure 2 provides a model of feedback to enhance learning as proposed by Hattie and Timperley (2007). An important feature is that a “pre-condition for effective feedback requires it to be conceived as information that is received rather than given” (Brooks et al., 2019a, p. 18). Another important feature is that the teacher devises feedback questions that can be answered by the learner, based on the visible learning technique of feeding up (Where am I going?), feeding back (How am I going?), and feeding forward (Where to next?) (Hattie and Timperley, 2007). To become an active participant in the learning process, the intention of what is being learned, the goals of the learning, and the criteria for successful accomplishment of the learning all need to be understood by the person who is “receiving” the learning; that is, learners themselves. When this occurs, teachers are able to observe self-regulated learning in action.

Another important aspect of visible learning occurs when the student provides feedback to the teacher. This is important because such feedback can be used by the teacher to adjust instruction and assess their own impact on the student's learning (Hattie, 2012; Brooks et al., 2019b). Thus, teachers need to create opportunities to get feedback from learners about the impact of their feedback and be open to receiving and acting upon this information. Research shows that this type of student to teacher feedback is most effective when it occurs within the learning period, rather than after learning (Hattie and Timperley, 2007).

A final feature of the relationships depicted in Figure 2 are the learning needs of the student. This is why Hattie and Timperley's (2007) model outlines the types of feedback that can be focused on how well tasks are understood or performed (Task Level), the main process needed to understand or perform the task (Process Level), the types of self-monitoring, direction and regulating of actions (Self-regulation Level), or the personal evaluations and affect about the learner (Self Level). As mentioned earlier, praise and criticism are often part of the Self Level (e.g., “You're fantastic at this”, “You can't do that”), but can diminish or even have a damaging effect on learning because they do not include information on what is being learned and tend therefore to be focused on aspects of the learner's personality (Hattie, 2009).

**Figure 3:**
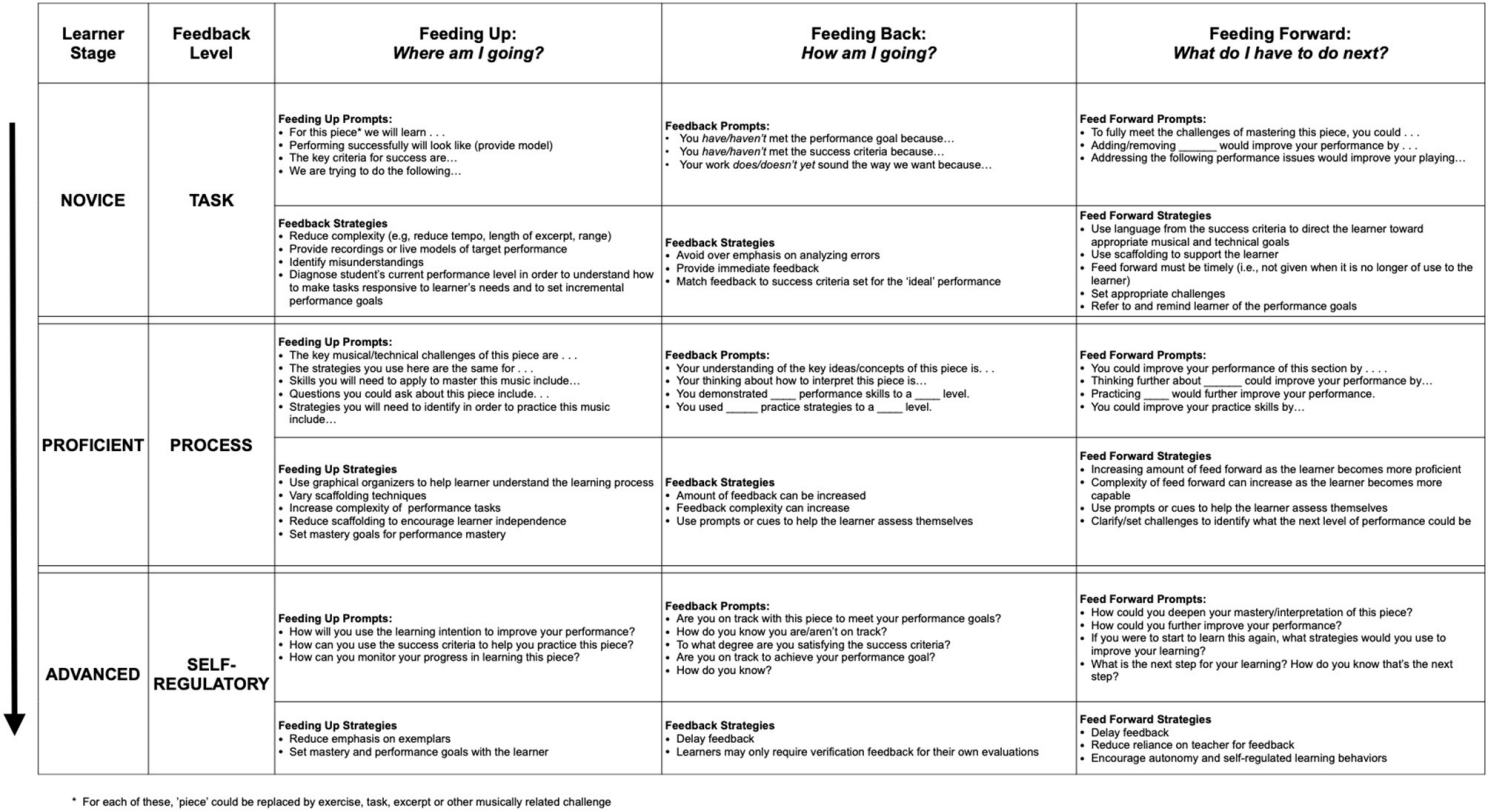
A matrix of feedback for learning. Adapted for music performance teaching from Brooks et al. (2019a, p. 28).

From their studies, Brooks et al. (2019a) devised a feedback matrix that seeks to not only provide a conceptual model of effective teacher feedback, but also a model that teachers can use to put these ideas into practice (see also, Hattie et al., 2017). Figure 3 is our adaptation of this perspective for studio teaching, which provides some guidance for how the feedback types and levels could be effectively used in instrumental and vocal teaching. We have provided both statements to give specific, actionable feedback to students, and questions to help make student learning more visible to both themselves and the teacher.

## Conclusions

Providing meaningful feedback to a music student is challenging and requires most music teachers to rethink the way they communicate with their students. With this in mind, there are at least seven general principles that can be followed for improving the quality of feedback:

- *Less monolog and more dialogue* (McPherson and Hattie, 2022). Feedback is a two-way conversation, and students need to be able to express confusion, alternative ideas, and their own musical ideas to become independent musicians.
- *Focus on improvement feedback*. Students need to know exactly what to do to improve errors, rather than focusing on the errors themselves.
- *Don't generalize*. Try to be specific with what needs to be practiced in order to achieve a goal.
- *Ask the learner to tell you* what they are doing, thinking, and feeling. These questions provide invaluable information to the teacher on how best to support the learner behaviorally, cognitively, and affectively.
- *Don't confuse feedback with praise*. While, learners will value and even seek praise, it does not provide meaningful information for learning and can dilute meaningful feedback. Teachers should be aware of providing feedback that is actionable during learning and reserve praise for developing rapport.
- *Check to see if your feedback is heard, understood, and actionable*. Just because we provide feedback does not mean it was heard, understood, and can led to actionable improvements by the student. If it is not actioned then it is likely to not be heard or understood, and questions the worthwhileness of the feedback we provide. It is always valuable to invite students to explain your feedback and talk together how they can action it.
- *Try another approach* if you feel the learner isn't responding, starts to become frustrated, or becomes critical of themselves. Careful scaffolding and incremental improvement are essential, especially given the evidence that successive failures are not helped by any type of feedback.

Another way of thinking about and refining one's use of feedback in learning situations is to focus on how to make comments that help focus the learner on the task being learned, the processes that might be adopted in order to master a task, the self-regulation strategies that could be adopted to implement these strategies, and the personal feelings that lead to a sense of accomplishment and progress along the journey to mastery. Some examples of these are shown in Table 2, where the emphasis is on shifting feedback comments so that they are not perceived by the learner as criticism or unspecified praise, but comments that target specific issues that need to address to get to the next level, set goals for themselves and increase an awareness of where to next.

**Table 2:** Examples of how to reshape feedback in music learning.

**Feedback about the…**	**Instead of…**	**Consider**
Task	That was great!	That was a major improvement—what differences did you notice? That was very different. Can you explain to me why it was so different?
Task	That didn't work.	When you phrased like *x*, I felt *y* or I didn't hear it
Task	You should play that section like this.	Here's another way to play that section. Which you do prefer? Why?
Process	Do X, Y, Z to improve this passage.	Walk me through how you would work on this passage. Why would you do it this way?
Self-regulation	Practice like this.	Here are some strategies to help you manage your practice time. Try them this week and we'll discuss how they worked for you.
Self-level	You're great!	You've worked hard on this! Walk me through your process for how you learned this.

Finally, in a book aimed at business leaders, Buckingham and Goodall (2019) argued that focusing people on their shortcomings impairs rather than enables performance. This is because humans are notoriously unreliable at assessing other humans, and thus being overly critical is not only detrimental to learning, but likely to be at least somewhat inaccurate. Because of all sorts of error, and in a situation where music is no different to any other area, it is literally impossible even for an expert or highly experienced musician to evaluate another musician with perfect validity and reliability (Manturzewska, 2011; McPherson and Schubert, 2022). So, while giving feedback is essential, this feedback should be constructive, focused on improvement, and delivered with the assumption that all student musicians are capable of improving.

We would add to this a final comment from Hattie and Timperley (2007) whose research shows that feedback is one of the most powerful factors for enhancing learning, and also that “Feedback can only build on something; it is of little use when there is no initial learning or surface information” (p. 104). With this in mind, we encourage educators to consider how their feedback can lead the learner to true understanding and the ability to provide feedback for themselves, rather than relying on evaluations that are unavoidably limited by an outsider's perspective. If we lead students to understand themselves and their musical goals, we are much better positioned to foster independent, confident musicians who can continue to grow in their craft beyond formal instruction.

## Author Contributions

GM conceived the initial idea for the paper. GM and JB wrote the majority of the paper. JH provided feedback and additional content in writing the paper. All authors contributed to the article and approved the submitted version.

## Conflict of Interest

The authors declare that the research was conducted in the absence of any commercial or financial relationships that could be construed as a potential conflict of interest.

## Publisher's Note

All claims expressed in this article are solely those of the authors and do not necessarily represent those of their affiliated organizations, or those of the publisher, the editors and the reviewers. Any product that may be evaluated in this article, or claim that may be made by its manufacturer, is not guaranteed or endorsed by the publisher.
